# Effects of the Protein Concentration and Quality in a Canned Diet on the Fecal Microbiota of Healthy Adult Cats

**DOI:** 10.3390/metabo12020105

**Published:** 2022-01-24

**Authors:** Nadine Paßlack, Louisa Verena Thies, Wilfried Vahjen, Jürgen Zentek

**Affiliations:** Institute of Animal Nutrition, Department of Veterinary Medicine, Freie Universität Berlin, 14195 Berlin, Germany; louisathies@web.de (L.V.T.); Wilfried.Vahjen@fu-berlin.de (W.V.); Juergen.Zentek@fu-berlin.de (J.Z.)

**Keywords:** feline, microbiota, protein concentration, protein quality, diet, feces, ammonium, short-chain fatty acids, 16S rDNA sequencing

## Abstract

Feline diets can markedly differ in their protein concentration and quality, which might also affect the intestinal microbiota of cats. In the present study, 6 canned diets, differing in their protein quality (high/low, achieved by varying amounts of meat and collagen-rich ingredients) and concentration (high quality/low quality: 36.2/36.7% in dry matter (DM), 43.3/45.0% in DM and 54.9/56.1% in DM), were fed to 10 healthy adult cats for 6 weeks each. At the end of the feeding periods, fecal samples were collected to analyze the microbiota (16S rDNA sequencing) and bacterial metabolites. Increasing dietary protein concentrations increased the relative abundance of *Fusobacterium* and *Bacteroides* as well as the concentrations of ammonium and n-valerate in the feces of the cats, independently of the dietary protein quality. A lower dietary protein quality was accompanied by a higher evenness index and a higher relative abundance of *Fusobacteria* and *Bacteroidetes* in the feces when compared to the feeding of the high protein quality diets. A promotion of bacterial proteolytic activity and, in particular, increased intestinal ammonium concentrations might be undesired effects of high protein intakes in cats. Whether the long-term feeding of those diets could be critical for feline health requires further investigation.

## 1. Introduction

As strict carnivores, cats usually prefer high protein/low carbohydrate diets [1]. Feline diets, however, can markedly differ in the protein concentration, and also in the protein sources used. These dietary variations could affect the protein digestibility and, as a consequence, also the intestinal microbiota, as varying amounts of bypass protein may enter the large intestine.

A few studies have already evaluated the effects of a high protein diet on the fecal microbiota of cats, as recently summarized by Pilla and Suchodolski [2]. As one finding, an increase in proteolytic bacteria, such as *Fusobacteria* or *Clostridium*, was often observed [3,4,5,6,7]. In these studies, different types of diets (canned, dry, raw) as well as varying dietary protein levels were evaluated. In addition, not only the protein concentrations of the experimental diets of one study differed, but also other nutrient concentrations that could potentially affect the intestinal microbiota, such as dietary fiber and fat [8]. The most recent study of Badri et al. [8] used feline dry extruded diets with increasing protein concentrations but comparable fat levels. The fiber concentrations, however, varied between the diets. The authors found a higher alpha-diversity of the fecal microbiota and a positive association with bacterial proteolytic function, amino acid, and urea metabolism as well as with bacterial mucin degradation at high protein intakes of the cats [8]. Interestingly, the apparent and true protein digestibility was higher when the cats received diets with the highest crude protein concentration (54.8% in dry matter (DM)) than with the lowest crude protein concentration (28.3% in DM). This effect was hypothesized to be a result of the higher protein or the lower dietary fiber intake, when the cats received the high protein diet [8].

A higher apparent crude protein digestibility at high protein intakes was also observed in our previous study evaluating dry extruded diets in cats [9], but not when canned diets were used [10]. These divergent results might be attributed to the different composition of dry extruded and canned pet food, particularly with regard to the higher amounts of cereals and fiber-rich ingredients in dry diets, or meat and meat by-products in canned diets, i.e., differences in nutrient interactions and the dietary protein sources. 

Considering the varying effects of high protein intakes on the apparent nutrient digestibility of cats when fed either a dry extruded diet [8,9] or a canned diet [10], a different impact on the intestinal microbiota can also be assumed. The present study therefore aimed to investigate the effects of increasing protein concentrations in a canned diet on the fecal microbiota of cats. We further focused on the protein quality in the diets, as it was expected that not only the dietary protein concentration but also the protein sources would affect the feline gut microbiota.

## 2. Results

### 2.1. Fecal Microbiota

Increasing dietary protein concentrations did not affect the alpha diversity of the fecal microbiota of the cats (Table 1). A lower dietary protein quality increased the evenness index (*p* = 0.015), although the difference was small in general. The richness and Shannon index were not affected by the protein quality in the diets. 

The effects of the diets on the relative abundance of dominant bacterial phyla in the feces of the cats are presented in Table 2. With increasing dietary protein concentrations, the relative abundance of *Fusobacteria* increased (*p* for protein concentration = 0.006). A lower protein quality in the diets was accompanied by a higher relative abundance of *Fusobacteria* and *Bacteroidetes* in the feces of the cats (*p* for protein quality = 0.040 and 0.008, respectively). The dietary protein quality also affected the relative abundance of *Proteobacteria* in the fecal samples, with the highest relative abundance at the medium protein concentration of the low protein quality diets (quadratic effect, *p* = 0.032), but the lowest relative abundance at the medium protein concentration of the high protein quality diets (quadratic effect, *p* = 0.022). 

On the genus level, the effects of the dietary protein concentration were more pronounced than of the dietary protein quality (Table 3). An increase in the relative abundance of *Fusobacterium*, *Bacteroides* and *Parabacteroides*, but a decrease of *Prevotella*, *Erysipelothrichaceae*, *Faecalibacterium,* and *Phascolarctobacterium* was observed with increasing dietary protein concentrations (linear contrasts, *p* < 0.005), independently of the dietary protein quality. The relative abundance of *Prevotella*, *Fusobacterium,* and *Ethanoligenes*, however, was higher, when the low protein quality diets were fed (*p* for protein quality < 0.05). The relative abundance of *Robinsoniella* increased with increasing protein concentrations in the low protein quality diets (*p* = 0.003), but decreased at higher protein concentrations in the high protein quality diets (*p* = 0.044).

Only small effects of the diets were observed, when bacterial groups and species were detected by qPCR (Table 4). The copy numbers of the *Clostridium coccoides* cluster XIVa in the feces of the cats were higher, when the low protein quality diets were fed (*p* = 0.011), and a quadratic effect was observed for *Clostridioides difficile*, with the highest copy number at the medium protein concentration of the low protein quality diets (*p* = 0.005).

### 2.2. Fecal Microbial Metabolites

The measurement of bacterial metabolites in the feces of the cats revealed effects of the dietary protein concentration, but not of the dietary protein quality (Table 5). The concentrations of ammonium, i-valeric acid, and n-valeric acid as well as the relative amounts (% of total short-chain fatty acids (SCFA)) of n-valeric acid increased, and the concentrations of histamine, cadaverine, and total biogenic amines as well as the relative amounts of propionic acid decreased with increasing protein concentrations in the diet (linear effects, *p* < 0.05).

## 3. Discussion

Feline canned diets often contain high amounts of meat and animal by-products. While meat is highly digestible for cats, collagen-rich animal by-products can be digested to a lesser extent [11]. In our previous study [10], we could demonstrate that neither the protein quality nor the protein concentration in a canned diet affected the apparent crude protein digestibility in cats. The determination of the apparent crude protein digestibility, however, implies limitations, especially the additional detection of endogenous nitrogen in the feces and the bacterial protein fermentation in the large intestine [12]. As our results further indicated a lower energy utilization from the low protein quality diets, as demonstrated by a higher feed intake, but similar body weights of the cats compared to the feeding of the high protein quality diets, we hypothesized that a microbial protein fermentation in the large intestine could have falsified the digestibility data [10]. Based on this hypothesis, we aimed to analyze the samples of our previous investigation in more detail. In the present study, we therefore focused on the composition and metabolic activity of the fecal microbiota, evaluating the impact of both the dietary protein quality and the protein concentration.

As a main finding, the effects of the protein concentration in the diets were more pronounced than of the dietary protein quality. A high protein intake increased the relative abundance of *Fusobacterium* and *Bacteroides* in the feces of the cats, two genera that show a proteolytic activity in the intestine [13]. As a consequence thereof, the fecal concentrations of ammonium and i-valerate, two relevant metabolites of the bacterial protein fermentation [14], were also increased by high dietary protein concentrations. Given the enhanced microbial protein degradation at high protein intakes, it can be hypothesized that a higher amount of bypass protein has entered the large intestine of the cats than at lower dietary protein concentrations. Although no differences in the apparent crude protein digestibility were observed among the dietary treatment groups in our previous study [10], as outlined above, the present data on the composition and metabolic activity of the fecal microbiota therefore indicate that the bacterial protein fermentation might have falsified the digestibility data obtained before.

Interestingly, the fecal concentrations of biogenic amines decreased with increasing dietary protein concentrations. Biogenic amines are metabolites of the bacterial amino acid degradation [14], and as such, increased intestinal concentrations would be expected by an enhanced microbial protein fermentation, as already demonstrated for cats in vitro [15]. The reason for the observed decrease in fecal biogenic amines at high protein intakes of the animals in the present study remains unclear at this stage. It can be speculated that biogenic amines might have been microbially degraded in the large intestine of the cats to some extent or that microbial decarboxylases have been inhibited by higher intestinal pH values, possibly resulting from the increasing intestinal ammonium concentrations at high protein intakes. These hypotheses, however, have to be further evaluated in future studies.

We hypothesized that a lower protein quality in the diets increases the amount of bypass protein in the large intestine of cats, resulting in enhanced microbial proteolytic activity. The data of the present study can partly confirm this hypothesis. We observed a higher evenness index and a promotion of the bacterial phyla *Fusobacteria* and *Bacteroidetes* in the feces of the cats, when diets with a lower protein quality were fed. Although bacterial groups of these phyla show proteolytic activity, and an increase in the relative abundance of *Fusobacterium* and *Prevotella* was also detected in the fecal samples, no further specific effects of a lower dietary protein quality could be observed on the genus level. In addition, the fecal concentrations of bacterial metabolites were unaffected by the protein quality in the experimental diets. Overall, the results of the present study indicate that the protein concentration in the diet more effectively influences the intestinal microbiota of cats than the dietary protein quality. Nevertheless, it should be considered that a lower protein quality in the diets was achieved by a higher amount of collagen-rich ingredients. Other protein sources, particularly plant-based proteins, can differ in their digestibilities in cats [16,17,18] and might therefore exert divergent effects on the intestinal microbiota as observed in the present investigation. This, however, should be evaluated in future studies.

With regard to the fecal bacterial groups promoted by a lower dietary protein quality, it should finally not go unmentioned that the copy numbers of the *Clostridium coccoides* cluster XIVa were increased by these diets. Several species are associated with this cluster and are considered to be beneficial for intestinal health, especially based on their production of butyrate, an important energy substrate for colonocytes and with further health-promoting effects [19]. Nevertheless, the differences between the dietary treatment groups were relatively small, and the butyrate concentrations in the feces of the cats were not increased, when the diets with the low protein quality were fed. In this context, however, it should be noted that there might have been a higher bacterial butyrate formation, but which was not detectable by an examination of feces. The fecal concentration is an indicator but not a proof of a higher microbial production, since an increased intestinal absorption could have been present at the same time. Overall, the potential beneficial effects of the higher numbers of the *Clostridium coccoides* cluster XIVa in the feces of the cats when feeding the low protein quality diets require further investigation. 

Although cats are strict carnivores and therefore adapted to high protein intakes [20], the detected effects on the composition of the fecal microbiota, and, in particular, on the fecal bacterial metabolites deriving from protein fermentation, indicate potential undesired effects of high protein diets. As also observed in other studies [8,15], the fecal concentrations of ammonium increased when the cats received the high protein diets. Ammonia is a toxic metabolite of bacterial protein fermentation and has to be converted to urea in the hepatic urea cycle and excreted by the kidneys afterwards [21]. Generally, the renal excretion of uremic toxins deriving from bacterial protein degradation, such as urea, can stress kidney function. While conflicting data exist on the potential role of dietary protein for the development of a chronic kidney disease in healthy cats [22,23], a moderate reduction of dietary protein in cats already suffering from kidney diseases is usually recommended [24,25]. The present study was conducted with healthy cats, and no detrimental effects of the diets on the kidney function of the animals could be detected based on the blood values measured [10]. However, given the increased concentrations of bacterial protein metabolites in the feces of the cats at high protein intakes, an adverse promotion of protein degradation in the large intestine of the animals can be concluded.

As a limitation of this study, the experimental diets did not only vary in their protein concentrations, but also to a small extent in the amounts of fiber. Since fibers can modulate the intestinal microbiota of cats, as reviewed by Pilla and Suchodolski [2], the dietary variations could have slightly affected the present results. However, the crude fiber concentrations in the diets were low in general, and the determination of total dietary fiber demonstrated values below the detection limit [10]. Thus, the observed effects on the fecal microbiota of the cats might be not a result of the varying fiber concentrations in the diets, but mainly attributed to the dietary protein concentrations and qualities.

## 4. Material and Methods

### 4.1. Study Design

Fecal samples obtained in the study described previously [10,26] were used for the present investigation. The study was approved by the Landesamt für Gesundheit und Soziales, Berlin, Germany (G 0138/12).

Ten healthy adult cats were fed six canned diets. The cats were physically examined by a veterinarian, and blood was analyzed for a complete blood count and clinical chemistry on a regular basis. The blood variables measured during the present study have been published previously [10,26]. The average body condition score (BCS) was 4–5/9, which is considered an ideal BCS in cats [27]. One cat was rated as thin (BCS of 3/9) and one cat as overweight (BCS 6/9).

Based on the amount of collagen-rich ingredients, the diets were considered high protein quality or low protein quality diets. Both the high and low protein quality diets were composed to achieve three different protein concentrations (36.2/36.7% in DM, 43.3/45.0% in DM, and 54.9/56.1% in DM, for the high and low protein quality diets, respectively). The dietary hydroxyproline concentrations were used as an indicator for the amount of collagen-rich ingredients in the diets (Table 6).

Each diet was fed for six weeks using a randomized cross-over design. The cats were fed individually, and the daily amount of feed was calculated to maintain body weight [28]. After a 31-day adaptation period, the cats were individually housed for 2 × 4 days, with a 3-day rest in between, to collect the feces of the animals.

### 4.2. Analysis of the Microbiota and Bacterial Metabolites in the Fecal Samples

The fecal microbiota was analyzed using 16S rDNA sequencing as described previously [29]. In short, the DNA extraction was performed using an extraction kit (QIAamp Fast DNA stool mini kit, Qiagen), following the instructions of the manufacturer but including a lysis step at 90 °C. For the sequencing, the V3-V4 region of the 16S rDNA gene was targeted. The final analysis was performed by the QIIME2 pipeline [30] and the SILVA SSU database [31]. Sequencing data with values < 0.5% in all groups are only reported in Tables if a significant group difference was detected.

In addition, selected bacterial groups and species were quantitatively detected in the feces of the cats by qPCR, using primers (Eurofins Genomics, Ebersberg, Germany) as specified in Table S1. The following PCR conditions were applied: 1 × 15 min. 95 °C; 40 × 15–30 s. 95 °C, 30–60 s. 48–63 °C, 30–60 s. 72 °C; 1 × 60 s. 25 °C. The Brilliant II SYBR^®^ Green Low ROX QPCR Master Mix (Agilent Technologies Inc., Santa Clara, CA, USA) as well as the real-time cycler Stratagene MX3000Pro (Agilent Technologies, Santa Clara, CA, USA) were used for the PCR amplification and data measurements.

The fecal concentrations of SCFA, ammonium, and lactate were measured by gas chromatography (Model 19095 N-123, Agilent Technologies, Santa Clara, CA, USA), colorimetrically (Berthelot reaction), and by high-performance liquid chromatography (HPLC Agilent 1100, Agilent Technologies, Santa Clara, CA, USA), respectively [32,33]. The concentrations of biogenic amines were measured in the feces using the Biochrom 20 Plus (Laborservice Onken GmbH, Gründau, Germany) [33].

### 4.3. Statistical Data Analysis

The data were analyzed with IBM^®^ SPSS 22 (SPSS Inc., Chicago, IL, USA) and are presented in Tables as means and the pooled standard error of the means (SEM). To evaluate the dietary effects on the fecal microbiota of the cats, a repeated-measures ANOVA was performed (within-subject factors: dietary protein concentration (3 levels) and dietary protein quality (2 levels)), and polynomial contrasts were calculated. In the case in which no interaction between the protein quality and concentration in the diet was present, the polynomial contrasts were calculated for the high and low protein quality groups together at the same protein level. If an interaction between the dietary protein concentration and quality was detected, the polynomial contrasts were separately calculated for the high and low protein quality groups. A *p* Value < 0.05 was considered to be statistically significant.

## 5. Conclusions

The protein concentration in a diet seems to be more effective to influence the fecal microbiota of cats than the dietary protein quality. Increasing dietary protein concentrations increased the relative abundance of proteolytic bacteria and the concentrations of metabolites deriving from microbial protein degradation in the feces of cats. A lower dietary protein quality also promoted proteolytic bacteria in the feces of the animals, although to a lesser extent. Overall, the present results indicate undesired effects of high protein intakes in cats, particularly with regard to shifts in the composition of the intestinal microbiota or increased intestinal ammonium concentrations. The relevance for feline health, however, warrants further investigation.

## Figures and Tables

**Table 1:** Alpha diversity of the fecal microbiota of cats (*n* = 10) fed diets with varying protein concentrations and qualities. Means and pooled standard error of the means (SEM).

	Low Protein Quality			High Protein Quality			SEM	*p* Value (Polynomial Contrasts)							
								Inter-action	Low Protein Quality		High Protein Quality		Protein Concentration		Protein Quality
	36.7	45.0	56.1	36.2	43.3	54.9			Lin ^1^	Quadr ^1^	Lin ^1^	Quadr ^1^	Lin ^1^	Quadr ^1^	
CHAO1 index	21.0	26.0	25.0	30.1	23.7	26.4	1.40	0.091	**-**	**-**	**-**	**-**	0.805	0.801	0.197
Shannon index	4.68	4.81	4.79	4.80	4.55	4.72	0.04	0.179	**-**	**-**	**-**	**-**	0.993	0.433	0.526
Evenness	0.56	0.56	0.55	0.54	0.53	0.55	0.00	0.597	**-**	**-**	**-**	**-**	0.707	0.401	0.015

^1^ Lin: linear; Quadr: quadratic.

**Table 2:** Relative abundance (%) of dominant bacterial phyla in the feces of cats fed diets with varying protein concentrations and qualities. Means and pooled standard error of the means (SEM).

	Low Protein Quality						High Protein Quality						SEM	*p* Value (Polynomial Contrasts)							
														Inter-action	Low Protein Quality		High Protein Quality		Protein Concentra-tion		Protein Quality
	36.7	*n* ^1^	45.0	*n*	56.1	*n*	36.2	*n*	43.3	*n*	54.9	*n*			Lin	Quadr	Lin	Quadr	Lin	Quadr	
*Actinobacteria*	7.68	10	3.87	10	8.15	10	5.75	10	6.94	10	6.93	10	0.50	0.138	-	-	-	-	0.429	0.168	0.979
*Bacteria* ^2^	1.27	10	3.49	10	1.57	10	2.09	10	0.91	10	1.41	10	0.33	0.064	-	-	-	-	0.800	0.497	0.366
*Bacteroidetes*	8.47	10	13.9	10	6.80	10	6.46	10	4.83	10	5.34	10	0.79	0.169	-	-	-	-	0.205	0.104	0.008
*Firmicutes*	79.8	10	71.1	10	75.1	10	82.5	10	84.3	10	79.2	10	1.12	0.102	-	-	-	-	0.092	0.633	<0.001
*Fusobacteria*	2.22	10	5.96	10	7.96	10	2.09	10	2.65	10	5.90	10	0.62	0.422	-	-	-	-	0.006	0.881	0.040
*Proteobacteria*	0.57	10	1.67	10	0.38	10	1.14	10	0.33	10	1.23	10	0.16	0.039	0.540	0.032	0.913	0.022	-	-	-

^1^ Number of positive samples; ^2^ not further specified domain; Lin: linear; Quadr: quadratic.

**Table 3:** Relative abundance (%) of dominant bacterial genera in the feces of cats fed diets with varying protein concentrations and qualities. Means and pooled standard error of the means (SEM).

	Low Protein Quality						High Protein Quality						SEM	*p* Value (Polynomial Contrasts)							
														Inter-action	Low Protein Quality		High Protein Quality		Protein Concentration		Protein Quality
	36.7	*n* ^1^	45.0	*n*	56.1	*n*	36.2	*n*	43.3	*n*	54.9	*n*			Lin	Quadr	Lin	Quadr	Lin	Quadr	
*Alistipes*	0.04	5	0.23	7	0.13	9	0.03	5	0.03	5	0.72	6	0.11	*	*	*	*	*	*	*	*
*Alkaliphilus*	1.52	10	0.97	10	1.15	10	0.42	9	2.76	10	0.69	10	0.34	0.285	-	-	-	-	0.900	0.495	0.904
*Anaerobiospirillum*	0.46	8	1.12	10	0.30	9	0.70	8	0.27	6	0.53	8	0.13	0.264	-	-	-	-	0.181	0.380	0.135
*Atopobium*	3.29	3	1.29	3	3.23	5	1.67	3	1.76	3	1.45	3	0.28	*	*	*	*	*	*	*	*
*Bacillus*	0.40	6	0.23	5	0.05	7	0.88	8	0.05	9	0.27	6	0.17	*	*	*	*	*	*	*	*
*Bacteria ^2^*	1.27	10	3.49	10	1.57	10	2.09	10	0.91	10	1.41	10	0.33	0.064	-	-	-	-	0.800	0.497	0.366
*Bacteroides*	1.10	10	3.49	10	3.18	10	2.13	10	1.76	8	2.67	10	0.29	0.289	-	-	-	-	0.020	0.320	0.475
*Blautia*	20.7	10	14.8	10	13.0	10	19.1	10	19.0	10	17.4	10	1.10	0.489	-	-	-	-	0.070	0.704	0.247
*Butyricicoccus*	1.12	10	1.08	9	1.59	10	0.78	9	0.88	10	0.89	10	0.10	0.071	-	-	-	-	0.110	0.539	0.099
*Butyrivibrio*	0.51	10	0.55	10	0.48	10	0.88	9	0.72	9	0.36	10	0.09	0.611	-	-	-	-	0.258	0.704	0.507
*Campylobacter*	0.02	2	0.68	5	0.13	3	0.05	3	0.01	2	0.01	1	0.20	*	*	*	*	*	*	*	*
*Clostridiales ^2^*	1.00	9	0.95	10	0.69	10	0.76	10	0.56	10	0.64	10	0.12	0.770	-	-	-	-	0.660	0.978	0.224
*Clostridium*	17.6	10	21.6	10	16.2	10	21.2	10	25.3	10	19.7	10	1.22	0.988	-	-	-	-	0.587	0.133	0.142
*Collinsella*	5.32	10	2.64	10	5.05	10	4.45	10	4.59	10	5.21	10	0.37	0.277	**-**	**-**	-	-	0.788	0.165	0.513
*Enterococcus*	0.19	9	0.28	10	0.14	10	0.40	10	0.10	9	0.61	6	0.07	0.389	**-**	**-**	-	-	0.719	0.524	0.559
*Ethanoligenens*	0.96	10	1.19	10	1.63	10	0.56	10	0.34	10	0.93	10	0.10	0.434	**-**	**-**	-	-	0.056	0.087	0.015
*Erysipelotrichaceae* ^2^	6.09	10	1.86	10	1.73	10	3.16	10	1.57	10	1.56	10	0.36	0.178	-	**-**	-	-	0.002	0.011	0.111
*Erysipelothrix*	1.35	6	0.83	4	0.87	7	0.66	6	1.23	5	0.46	5	0.14	0.388	-	**-**	-	-	0.966	0.925	0.334
*Eubacterium*	3.95	10	4.10	10	5.11	10	2.66	10	2.75	10	4.36	10	0.34	0.855	-	**-**	-	-	0.138	0.447	0.119
*Faecalibacterium*	2.44	10	1.18	10	0.95	10	2.00	10	0.82	10	0.53	10	0.15	0.960	-	-	-	-	0.001	0.119	0.166
*Fusobacterium*	2.22	10	5.96	10	7.96	10	2.09	10	2.65	10	5.90	10	0.62	0.422	-	-	-	-	0.006	0.881	0.039
*Gordonibacter*	1.18	10	0.70	10	1.16	10	0.43	10	1.71	10	0.86	10	0.21	0.263	-	-	-	-	0.711	0.516	0.965
*Hespellia*	0.61	7	0.87	6	1.35	10	1.53	8	0.16	8	0.46	7	0.20	0.147	-	-	-	-	0.808	0.107	0.634
*Lachnospiraceae* ^2^	0.17	6	0.96	7	0.34	8	0.71	5	0.29	8	0.48	5	0.11	*	*	*	*	*	*	*	*
*Lactococcus*	0.61	6	0.19	7	1.17	7	1.23	8	2.39	8	0.75	9	0.25	0.554	-	-	-	-	0.231	0.143	0.908
*Leuconostoc*	0.88	6	0.16	1	0.05	3	0.53	6	0.68	6	2.08	3	0.30	*	*	*	*	*	*	*	*
*Megamonas*	0.76	7	0.29	8	0.11	6	0.06	6	0.10	4	0.10	4	0.12	*	*	*	*	*	*	*	*
*Oxobacter*	-	0	1.49	1	0.02	2	0.06	5	0.11	2	0.69	2	0.15	*	*	*	*	*	*	*	*
*Parabacteroides*	0.11	10	0.32	10	0.19	10	0.22	9	0.21	7	0.28	9	0.04	0.649	-	-	-	-	0.029	0.167	0.537
*Peptococcus*	0.49	6	0.77	6	1.17	9	1.22	9	0.94	10	1.15	8	0.14	0.910	-	-	-	-	0.702	0.759	0.443
*Peptostreptococca-* *ceae* ^2^	10.0	10	9.30	10	13.8	10	12.5	10	14.2	10	15.4	10	0.86	0.647	-	-	-	-	0.097	0.555	0.089
*Peptostreptococcus*	1.53	5	0.23	8	0.13	6	2.19	7	0.03	7	0.09	6	0.43	*	*	*	*	*	*	*	*
*Phascolarcto-* *bacterium*	0.68	10	0.60	10	0.33	10	0.54	10	0.20	10	0.24	10	0.07	0.513	-	-	-	-	0.004	0.767	0.183
*Prevotella*	6.99	10	9.36	10	2.87	10	3.81	10	2.91	10	1.63	10	0.62	0.160	-	-	-	-	0.011	0.064	0.011
*Propionibacterium*	0.05	2	0.22	2	0.04	3	0.03	1	0.04	3	0.53	1	0.05	*	*	*	*	*	*	*	*
*Raoultella*	0.19	1	0.01	1	0.07	1	1.62	2	0.15	4	1.62	1	0.33	*	*	*	*	*	*	*	*
*Robinsoniella*	1.98	10	2.50	10	5.12	10	3.64	10	4.11	10	1.96	10	0.36	0.031	0.003	0.240	0.044	0.368	-	-	-
*Roseburia*	2.30	10	3.19	10	3.66	10	2.98	10	2.75	9	3.10	10	0.36	0.738	-	-	-	-	0.463	0.863	0.570
*Ruminococcus*	1.61	10	1.48	10	1.75	10	1.13	10	1.84	9	1.79	10	0.16	0.584	-	-	-	-	0.355	0.720	0.679
*Selenomonas*	2.82	8	2.46	10	2.91	9	2.39	10	2.62	8	3.04	10	0.22	0.454	-	-	-	-	0.746	0.434	0.979
*Vagococcus*	0.03	6	0.19	2	0.73	3	0.07	7	0.05	8	4.04	6	0.74	*	*	*	*	*	*	*	*

^1^ Number of positive samples; ^2^ Not further specified domain, bacterial class, order, or family; Lin: linear; Quadr: quadratic; * polynomial contrasts could not be calculated.

**Table 4:** Copy numbers (log_10_/g) of bacterial groups and species in the feces of cats fed diets with varying protein concentrations and qualities. Means and pooled standard error of the means (SEM).

	Low Protein Quality						High Protein Quality						SEM	*p* Value (Polynomial Contrasts)							
														Inter-action	Low Protein Quality		High Protein Quality		Protein Concentra-tion		Protein Quali-ty
	36.7	*n* ^1^	45.0	*n*	56.1	*n*	36.2	*n*	43.3	*n*	54.9	*n*			Lin	Quadr	Lin	Quadr	Lin	Quadr	
*Bacteroides/* *Prevotella*/ *Porphyromonas*	10.5	10	10.8	10	10.2	10	10.2	10	9.83	10	10.1	10	0.08	0.016	0.114	0.119	0.829	0.174	-	-	-
*Bifidobacterium* spp.	7.63	3	7.21	5	6.72	6	6.90	4	6.82	2	6.84	3	0.10	*	*	*	*	*	*	*	*
*Clostridioides difficile*	4.62	9	5.75	10	5.36	8	5.64	10	5.17	9	5.27	10	0.11	0.010	0.209	0.005	0.357	0.435	-	-	-
*Clostridium* *cluster I*	9.51	10	9.95	10	9.57	10	9.35	10	9.48	10	9.55	10	0.06	0.217	-	-	-	-	0.266	0.104	0.068
*Clostridium coccoides* cluster XIVa	10.0	10	10.4	10	9.91	10	9.68	10	9.61	10	9.86	10	0.08	0.105	-	-	-	-	0.801	0.488	0.011
*Clostridium* *leptum* cluster IV	9.36	10	9.60	10	9.10	10	8.97	10	8.71	10	8.93	10	0.08	0.032	0.166	0.111	0.842	0.317	-	-	-
*Clostridium perfringens*	9.25	10	9.54	10	9.32	10	9.15	10	9.33	10	9.28	10	0.05	0.605	-	-	-	-	0.308	0.079	0.215
*Enterobacteria-ceae*	7.74	10	7.84	10	7.31	8	7.36	8	7.50	8	7.52	9	0.06	0.151	-	-	-	-	0.776	0.113	0.112
*Escherichia coli/* *Hafnia/Shigella*	8.16	8	8.15	5	6.91	3	7.24	8	7.61	4	7.24	6	0.13	*	*	*	*	*	*	*	*
*Lactobacillus* spp.	7.98	8	6.76	9	6.47	9	7.35	8	7.31	10	7.07	7	0.17	0.394	-	-	-	-	0.429	0.849	0.509
*Salmonella* spp.	-	1	5.04	6	4.74	2	-	1	-	1	4.66	2	0.11	*	*	*	*	*	*	*	*

^1^ Number of positive samples; Lin: linear; Quadr: quadratic; * Polynomial contrasts could not be calculated.

**Table 5:** Concentrations of bacterial metabolites in the feces of cats fed diets with varying protein concentrations and qualities. Means and pooled standard error of the means (SEM).

	Low ProteinQuality			High Protein Quality			SEM	*p* Value (Polynomial Contrasts)							
								Inter-action	Low Protein Quality		High Protein Quality		Protein Concentration		Protein Quality
	36.7	45.0	56.1	36.2	43.3	54.9			Lin	Quadr	Lin	Quadr	Lin	Quadr	
µmol/g															
Ammonium	48.3	54.1	75.3	47.7	52.5	75.2	3.64	0.972	-	-	-	-	0.030	0.086	0.906
L-lactate	0.25	0.17	0.13	0.12	0.12	0.20	0.02	0.334	-	-	-	-	0.719	0.316	0.382
D-lactate	0.03	0.01	0.02	0.01	0.02	0.01	0.00	0.536	-	-	-	-	0.691	0.732	0.415
Acetic acid	54.9	60.6	77.3	59.7	60.3	71.5	4.45	0.809	-	-	-	-	0.086	0.335	0.971
Propionic acid	21.3	21.5	23.2	24.3	20.9	23.0	1.26	0.674	-	-	-	-	0.909	0.309	0.843
i-butyric acid	3.63	4.06	4.28	3.66	3.84	5.01	0.21	0.485	-	-	-	-	0.089	0.522	0.674
n-butyric acid	13.3	14.5	15.0	13.6	12.8	15.2	0.71	0.679	-	-	-	-	0.249	0.675	0.844
i-valeric acid	6.62	7.35	8.43	6.62	6.94	9.12	0.35	0.630	-	-	-	-	0.043	0.242	0.869
n-valeric acid	5.14	6.23	6.80	4.86	5.33	6.83	0.28	0.656	-	-	-	-	0.020	0.777	0.602
Total SCFA	105	114	135	113	110	131	6.74	0.831	-	-	-	-	0.108	0.329	0.992
% of total SCFA															
Acetic acid	49.7	52.1	54.7	51.1	53.1	52.8	1.08	0.765	**-**	**-**	**-**	**-**	0.143	0.726	0.917
Propionic acid	20.3	19.0	17.5	21.4	18.7	17.6	0.29	0.378	-	-	-	-	<0.001	0.319	0.391
i-butyric acid	3.65	3.64	3.37	3.46	3.75	4.02	0.12	0.149	-	-	-	-	0.572	0.685	0.422
n-butyric acid	14.1	13.0	11.9	12.9	12.3	12.5	0.56	0.838	-	-	-	-	0.322	0.817	0.473
i-valeric acid	6.91	6.65	6.84	6.45	6.87	7.50	0.27	0.520	-	-	-	-	0.287	0.637	0.801
n-valeric acid	5.30	5.67	5.69	4.66	5.29	5.65	0.23	0.850	-	-	-	-	0.033	0.601	0.126
µmol/g															
Putrescine	0.34	0.19	0.34	0.96	0.19	0.11	0.08	0.145	-	-	-	-	0.056	0.098	0.258
Histamine	0.53	0.25	0.08	0.24	0.18	0.12	0.04	0.088	-	-	-	-	0.024	0.367	0.118
Cadaverine	2.17	2.12	1.41	2.48	1.80	0.93	0.20	0.619	-	-	-	-	0.025	0.471	0.529
Spermidine	0.36	0.33	0.33	0.45	0.39	0.32	0.02	0.605	-	-	-	-	0.165	0.997	0.345
Tyramine	0.08	0.15	0.06	0.08	0.08	0.11	0.02	0.523	-	-	-	-	0.871	0.565	0.955
Spermine	0.01	0.01	0.01	0.01	0.01	0.01	0.00	0.985	-	-	-	-	0.558	0.426	0.181
Total biogenic amines	3.50	3.06	2.23	4.22	2.66	1.60	0.30	0.512	-	-	-	-	0.017	0.945	0.768

Lin: linear; Quadr: quadratic; SCFA: Short-chain fatty acids.

**Table 6:** Analyzed dry matter (DM) and nutrient concentrations of the experimental diets [10,26].

	Low Protein Quality, Protein Concentration ^1^			High Protein Quality, Protein Concentration ^2^		
	36.7%	45.0%	56.1%	36.2%	43.3%	54.9%
DM (g/kg)	200	189	204	216	203	193
g/kg DM						
Crude protein	367	450	561	362	433	549
Hydroxyproline	3.76	8.45	9.44	2.56	3.76	4.45
Crude fat	284	296	271	294	318	262
Crude fiber ^3^	3.14	7.05	10.5	6.75	4.46	6.78
Crude ash	77.2	73.8	83.4	68.3	68.9	75.2
Nitrogen-free extracts (calculated)	269	173	74.7	269	176	107
Calcium	11.2	11.4	12.6	10.7	11.2	11.8
Phosphorus	8.79	8.34	8.88	7.73	8.23	8.71
Sodium	5.83	5.75	8.20	4.88	5.78	6.74
Potassium	11.1	9.35	12.4	8.75	8.58	8.67
Magnesium	0.48	0.48	0.47	0.45	0.47	0.49

^1^ Ingredients in descending order: liver, trachea, wheat flour, meat, sunflower oil, greaves meal, blood meal, minerals, gelling and thickening agents, vitamins. ^2^ Ingredients in descending order: liver, meat, wheat flour, sunflower oil, blood meal, greaves meal, minerals, gelling and thickening agents, vitamins. ^3^ The total dietary fiber concentrations were below the detection limit in all experimental diets (Total Dietary Fiber Assay Kit, Megazyme u.c., Ireland).

## Data Availability

The sequencing data are available at the BioProject ID: PRJNA789745.

## Supplementary Materials

The following supporting information can be downloaded at https://www.mdpi.com/article/10.3390/metabo12020105/s1: Table S1: Primers used for the qPCR to quantitatively detect selected bacterial groups and species in the feces of cats fed a diet with varying protein concentrations and qualities.
