# Supplementary material for: Effects of the Protein Concentration and Quality in a Canned Diet on the Fecal Microbiota of Healthy Adult Cats
Source: Metabolites. 2022 Jan 24;12(2):105. doi: 10.3390/metabo12020105 (PMC8874684; doi:10.3390/metabo12020105)
Supplement: Supplementary file 1 [file metabolites-12-00105-s001.zip › metabolites-1566194-supplementary.pdf]

Table S1: Primers used for the qPCR to quantitatively detect selected bacterial groups and species in the feces of cats fed diets with varying protein concentrations and qualities.

Bacterial group/species	Primer	Sequence	Base pairs	Temperature (°C)	Reference
Bacteroides/Prevotella/Porphyromonas	BPP1<br>BPP2	5'-GGT GTC GGC TTA AGT GCC AT-3'<br>5'-CGG AYG TAA GGG CCG TGC-3'	147	55	[1]
Bifidobacterium spp.	g-BIFID-F<br>g-BIFID-R	5'-TCG CGT CYG GTG TGA AAG-3'<br>5'-CCA CAT CCA GCR TCC AC-3'	251	58	[1]
Clostridium coccoides cluster XIVa	g-Ccoc-F<br>g-Ccoc-R	5'-AAA TGA CGG TAC CTG ACT AA-3'<br>5'-CTT TGA GTT TCA TTC TTG CGA A-3'	455	60	[2]
Clostridium cluster I	Cl-F1<br>Cl-R2	5'-TAC CHR AGG AGG AAG CCA C-3'<br>5'-GTT CTT CCT AAT CTC TAC GCA T-3'	239	63	[3]
Clostridioides difficile	Cdiff-16S-1f<br>Cdiff-16S-1r	5'-TTG AGC GAT TTA CTT CGG TAA AGA-3'<br>5'-CCA TCC TGT ACT GGC TCA CCT-3'	174	58	Developed in the Institute of Animal Nutrition, Freie Universität Berlin
Clostridium leptum cluster IV	sg-Clept-F<br>sg-Clept-R	5'-GCA CAA GCA GTG GAG T-3'<br>5'-CTT CCT CCG TTT TGT CAA-3'	253	60	[4]
Clostridium perfringens	Cpa1a<br>Cpa1b	5'-AGG CGC TTA TTT GTG CTA CG-3'<br>5'-TCA ATC TTT CCA TCC CAA GC-3'	90	48	Developed in the Institute of Animal Nutrition, Freie Universität Berlin
Enterobacteriaceae	EntqPCR3417f<br>EntqPCR3852r	5'-GTB TCD CCR CGC AGR C-3'<br>5'-TGC GYC TGG TRA TCT A-3'	454	55	Developed in the Institute of Animal Nutrition, Freie Universität Berlin
Escherichia coli/Hafnia/Shigella	Entero-F<br>Entero-R	5'-GTT AAT ACC TTT GCT CAT TGA-3'<br>5'-ACC AGG GTA TCT AAT CCT GTT-3'	354	55	[5]
***Lactobacillus* spp.**	Lac1<br>Lac2	5'-AGC AGT AGG GAA TCT TCC A-3'<br>5'-CAC CGC TAC ACA TGG AG-3'	351	58	[1]
***Salmonella* spp.**	QVR133<br>QVR134	5'-GAA GCA GCG CCT GTA AAA TC-3'<br>5'-TGG CTG TGG TGC AAA ATA TC-3'	20	60	Developed in the Institute of Animal Nutrition, Freie Universität Berlin

References cited in this Supplementary Material

1. Rinttilä, T.; Kassinen, A.; Malinen, E.; Krogius, L.; Palva, A. Development of an extensive set of 16S rDNA-targeted primers for quantification of pathogenic and indigenous bacteria in faecal samples by real-time PCR. *J. Appl. Microbiol.* **2004**, *97*, 1166–1177.
2. Matsuki, T.; Watanabe, K.; Fujimoto, J.; Miyamoto, Y.; Takada, T.; Matsumoto, K.; Oyaizu, H.; Tanaka, R. Development of 16S rRNA-gene-targeted group-specific primers for the detection and identification of predominant bacteria in human feces. *Appl. Environ. Microbiol.* **2002**, *68*, 5445–5451.
3. Song, Y.; Liu, C.; Finegold, S.M. Real-Time PCR quantitation of clostridia in feces of autistic children. *Appl. Environ. Microbiol.* **2004**, *70*, 6459–6465.
4. Matsuki, T.; Watanabe, K.; Fujimoto, J.; Takada, T.; Tanaka, R. Use of 16S rRNA gene-targeted group-specific primers for real-time PCR analysis of predominant bacteria in human feces. *Appl. Environ. Microbiol.* **2004**, *70*, 7220–7228.
5. Malinen, E.; Kassinen, A.; Rinttilä, T.; Palva, A. Comparison of real-time PCR with SYBR Green I or 5′-nuclease assays and dot-blot hybridization with rDNA-targeted oligonucleotide probes in quantification of selected faecal bacteria. *Microbiology* **2003**, *149*, 269–277.
